# Electrochemical Detection of miR-29a and miR-34a Using AuNPs Immobilized by a Silsesquioxane Polyelectrolyte: Potential Early Alzheimer’s Disease Biomarkers Detection

**DOI:** 10.3390/s26072089

**Published:** 2026-03-27

**Authors:** Amanda Loos Vargas Zinser, Felipe Zahrebelnei, João Paulo Winiarski, Paulo Henrique de Souza Picciani, Karen Wohnrath, Christiana Andrade Pessôa

**Affiliations:** 1 Department of Chemistry, State University of Ponta Grossa, Ponta Grossa CEP 84030-900, PR, Brazil; 3100122007009@uepg.br (A.L.V.Z.); felipe.zahrebelnei@ifpr.edu.br (F.Z.); kawoh@uepg.br (K.W.); 2 Telêmaco Borba Campus, Federal Institute of Paraná, Telêmaco Borba CEP 84271-120, PR, Brazil; 3 Department of Chemistry, Federal University of Santa Catarina, Florianópolis CEP 88040-900, SC, Brazil; joao.winiarski@ufsc.br; 4 Institute of Macromolecules, Federal University of Rio de Janeiro, Rio de Janeiro CEP 21941-901, RJ, Brazil; picciani@ima.ufrj.br

**Keywords:** Alzheimer’s disease, electrochemical biosensor, miRNA-29a, miRNA-34a, silsesquioxane, gold nanoparticles

## Abstract

Alzheimer’s Disease (AD) is the leading cause of dementia worldwide, and early diagnosis is crucial to minimize neurological damage and loss of quality of life. Here, we report an electrochemical biosensor for detecting miRNAs 29a and 34a, potential non-invasive biomarkers associated with AD. The biosensor consisted of a glassy carbon electrode (GCE) modified with a novel nanohybrid of gold nanoparticles stabilized by 3-n-propyl(4-dimethylaminopyridinium) silsesquioxane chloride (AuNPs–Si4DMAP^+^Cl^−^). Thiolated anti-miRNA probes were immobilized separately on the GCE/AuNPs-Si4DMAP^+^Cl^−^, followed by BSA blocking. Target miRNAs were detected via hybridization with complementary probes using electrochemical impedance spectroscopy. The nanohybrid, characterized by spectroscopic and morphological techniques, significantly enhanced the electrochemical response and was effective detecting both miRNAs, showing suspension stability over 600 days. LOD and LOQ were 1.79 pM and 5.87 pM for miRNA-29a, and 2.21 pM and 11.01 pM for miRNA-34a. These results highlight the platform’s potential for electrochemical detection of these miRNAs in blood, supporting earlier detection of AD and other neurodegenerative diseases.

## 1. Introduction

Alzheimer’s disease (AD) is a progressive and debilitating condition that primarily affects neurons, leading to significant cognitive impairment due to tissue loss and neuronal cell death [1]. AD is the leading cause of dementia and according to the World Health Organization (WHO), approximately 10 million new cases of dementia are diagnosed globally each year, with up to 70% attributed to AD [2]. Studies suggest that the pathophysiological processes of AD begin at least a decade before the onset of clinical symptoms [3], indicating that early detection significantly improves treatment outcomes [4]. The deposition of β-amyloid (Aβ) is considered the first pathological hallmark of the preclinical stage of AD, followed by the formation of neurofibrillary tangles due to the aggregation of hyperphosphorylated tau protein (p-tau), which results in neurodegeneration, synaptic dysfunction and neuronal loss [5,6]. Among the most frequently reported in vivo biomarkers for AD are hyperphosphorylated tau (p-tau) and β-amyloid peptides (Aβ42, Aβ42/Aβ40) [7]. Although cerebrospinal fluid (CSF) sampling via lumbar puncture remains one of the most widely used methods for monitoring biomarker changes, clinical applications require biomarkers to be detectable in more accessible biological fluids, using safer and less invasive approaches.

Although the full spectrum of mechanisms underlying neurodegenerative diseases (NDs) remains unclear, deregulated expression of specific microRNAs (miRNAs) has been observed in several NDs, including AD [8]. miRNAs are small non-coding RNA sequences that regulate gene expression and are involved in the majority of biological processes [9,10]. The greatest advantage of miRNAs as potential biomarkers for NDs lies in their detectability in circulating biological fluids such as blood and urine, enabling more accurate, faster and minimally invasive diagnostics.

Studies have highlighted the relevance of miR-29a and miR-34a in AD progression, as they both can regulate β-secretase (BACE1) [11,12,13,14], the enzyme responsible for Aβ peptide production. A reduction in plasma miR-34a levels has been reported in AD patients [15] and its downregulation has been associated with increased Aβ-induced oxidative stress [16]. Furthermore, miRNA-34a is among the miRNAs suggested to exhibit dysregulation up to 20 years prior to the onset of clinical symptoms. miRNA-29a may be involved in key pathological processes associated with AD, including tau pathology, amyloid accumulation, neuroinflammation and amyloid precursor protein (APP) processing [17]. In addition, miRNA-29a-3p has been reported to be downregulated in the plasma of patients with Alzheimer’s disease [18]. These findings support the potential of miR-29a and miR-34a as valuable biomarkers for AD.

The development of electrochemical devices for detecting biomarkers associated with NDs, especially AD, is promising regarding cost, response time and sensitivity of the technique [19]. The work developed by Miglione et al. (2022) [20] describes the miRNA-29a detection using a screen-printed carbon electrode (SPCE) modified with Ag/AgCl and carbon conductive inks, onto which AuNPs and a methylene blue-labeled anti-miR-29a probe were immobilized, achieving a limit of detection (LOD) of 0.15 nM in solution and 0.2 nM in serum. Congur, Eksin, and Erdem (2018) [21] developed an impedimetric DNA biosensor for miRNA-34a detection using a graphite pencil electrode (PGE) modified with graphene oxide (GO), onto which an amine-functionalized DNA probe was immobilized. The hybridization event was monitored via electrochemical impedance spectroscopy (EIS) and the biosensor showed a LOD of 72 nM in diluted serum. More recently, Pereira et al. (2023) [22] developed a biosensor for miRNA-34a detection using SPCEs modified with electrodeposited AuNPs. The platform was functionalized with an anti-miRNA-34a probe and exhibited a LOD of 94 aM in diluted serum.

The modification of biosensors with AuNPs to enhance electrochemical response and platform sensitivity has been extensively reported due to the high electrical conductivity and favorable surface properties of AuNPs. Silsesquioxane-based polyelectrolytes exhibit positive charges along their polymeric chains, while their silica–oxygen frameworks act as stabilizing agents for various nanomaterials [23]. As a result, there is growing interest in developing silsesquioxane-based nanocomposites and films [24,25]. These materials have demonstrated potential as stabilizing agents in the synthesis of metallic nanoparticles (MNPs), aiming to obtain more stable nanocomposites and prevent aggregation, as well as in the formation of thin films for application in sensors and biosensors [26,27,28].

Therefore, aiming for less invasive and more sensitive detection with shorter analysis times, this study proposes the development of electrochemical devices based on glassy carbon electrodes (GCEs) modified with AuNPs stabilized in a silsesquioxane matrix for the detection of miRNA-29a and miRNA-34a as potential biomarkers for a minimally invasive and complementary approach to Alzheimer’s disease diagnostics.

## 2. Materials and Methods

### 2.1. Reagents and miRNA Oligonucleotides

The reagents sodium chloride (NaCl), monosodium phosphate (Na_2_HPO_4_) and disodium phosphate (NaH_2_PO_4_) used for the preparation of the phosphate-buffered saline (PBS) were purchased from Biosynth (Staad, Switzerland). Potassium ferrocyanide trihydrate (K_4_[Fe(CN)_6_]·3H_2_O), potassium ferricyanide (K_3_[Fe(CN)_6_]), chloroauric acid trihydrate (HAuCl_4_·3H_2_O) and bovine serum albumin (BSA) were obtained from Sigma-Aldrich (St. Louis, MO, USA). The compound 4-(dimethylamino)pyridine (4-DMAP) was acquired from Merck (Darmstadt, Germany). All solutions were prepared using high-purity Milli-Q water (resistivity 18.2 MΩ cm).

The miRNAs and anti-miRNAs were synthesized by Genone Biotechnologies and included: (i) a 5′-thiolated anti-miR-29a probe with the sequence 5′-C6-SH/TAACCGATTTCAGATGGTGCTA-3′; (ii) a non-thiolated target oligonucleotide complementary to anti-miR-29a: 5′-TAGCACCATCTGAAATCGGTTA-3′; (iii) a 5′-thiolated anti-miR-34a probe with the sequence 5′-C6-SH/ACAACCAGCTAAGACACTGCCA-3′; (iv) a non-thiolated target oligonucleotide complementary to anti-miR-34a: 5′-TGGCAGTGTCTTAGCTGGTTGT-3′. All oligonucleotide solutions were stored at −20 °C after preparation.

### 2.2. Synthesis and Characterization of AuNPs-Si4DMAP^+^Cl^−^ Nanohybrid

The synthesis of the silsesquioxane Si4DMAP^+^Cl^−^ from the organo-functionalization of the xerogel with 4-DMAP was carried out following the procedure reported by Winiarski et al. (2020) [29] and the synthesis of the nanohybrid was performed according to the protocol described in the literature for the preparation of AuNPs stabilized in a structurally similar silsesquioxane, as reported by Lima et al. (2022) [30]. The resulting polyelectrolyte and nanohybrid were characterized using FTIR (DRS-8000/SHIMADZU, model IRPrestige-21), UV-Vis spectroscopy (Varian, model Cary 50 spectrophotometer), zeta potential and dynamic light scattering (DLS) (Malvern/Zetasizer Nano ZS90, Zetasizer software 8.02). FTIR analyses were performed in the range of 400 to 4000 cm ^−1^ (resolution: 4 cm^−1^; number of scans: 64), with the Si4DMAP^+^Cl^−^ and AuNPs-Si4DMAP^+^Cl^−^ samples drop-cast onto potassium bromide (KBr), which was mixed and dried to form pellets for analysis. UV-Vis spectra were obtained in the range of 200 to 800 nm from aqueous diluted suspensions. For both materials, field-emission scanning electron microscopy (FEG-SEM) images were acquired using a scanning electron microscope (Tescan MIRA3) on plastic disk substrates coated with vitreous carbon, where the samples were drop-cast and subsequently metallized. Transmission electron microscopy (TEM) analysis of the AuNPs–Si4DMAP^+^Cl^−^ nanohybrid was also performed, using a suspension deposited onto Formvar-coated 300 mesh copper grids, dried, and imaged using a JEOL 2100F instrument operated at 200 kV.

### 2.3. Modified GCE Characterization and Preparation of miRNA Biosensor

**Figure 1:**
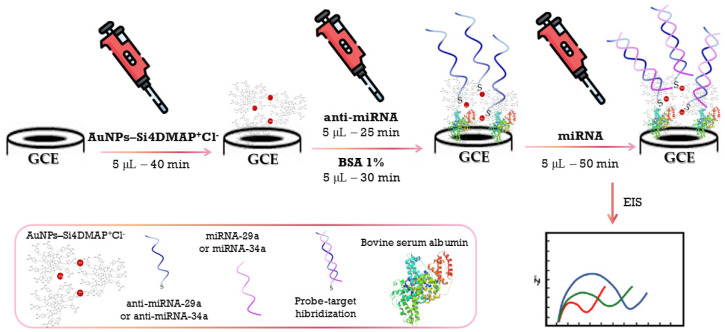
Schematic representation of the biosensor construction.

The surface of the glassy carbon electrode (GCE) was polished using a metallographic polishing cloth and alumina slurry with particle sizes of 1.0 μm, 0.5 μm, and 0.3 μm, respectively. After polishing, the electrode was rinsed with distilled water. The AuNPs–Si4DMAP^+^Cl^−^ nanohybrid was applied onto the GCE surface via the drop-coating technique by depositing 5.0 µL of the suspension and allowing it to dry for 40 min at room temperature, yielding the modified electrode GCE/AuNPs–Si4DMAP^+^Cl^−^. To facilitate the immobilization of the anti-miR-29a and anti-miR-34a probes without the need for more labor-intensive techniques, the oligonucleotides were obtained with a thiol group at the 5′ end, enabling covalent bonding to the AuNPs present on the nanohybrid-modified surface. The biosensor construction is illustrated schematically in Figure 1.

Probe immobilization was carried out by drop-casting 5.0 µL of a 10 pM solution of either anti-miRNA-29a or anti-miRNA-34a onto two separate modified GCEs, followed by incubation for 25 min in a humid chamber to promote thiol–gold covalent bonding. After incubation, the electrodes were rinsed with PBS buffer to remove unbound oligonucleotides, resulting in GCE/AuNPs–Si4DMAP^+^Cl^−^/anti-miRNA-29a and GCE/AuNPs–Si4DMAP^+^Cl^−^/anti-miRNA-34a. This step was followed by the immobilization of bovine serum albumin (BSA, 1%) solution prepared in PBS buffer. The electrodes were kept in a humid chamber for 30 min to block non-specific binding sites, then rinsed with PBS buffer to complete the biosensor architecture. The resulting systems were denoted as GCE/AuNPs–Si4DMAP^+^Cl^−^/probe/BSA (where probe = anti-miRNA-29a or anti-miRNA-34a). Subsequently, the respective target sequences (miRNA-29a and miRNA-34a) were drop-cast onto the biosensor surfaces to allow hybridization with the complementary probes. The electrodes were incubated for 50 min in a humid chamber, followed by rinsing in PBS. The final biosensor configurations were denoted as GCE/AuNPs–Si4DMAP^+^Cl^−^/anti-miRNA-29a/BSA/miRNA-29a and GCE/AuNPs–Si4DMAP^+^Cl^−^/anti-miRNA-34a/BSA/miRNA-34a, which were then subjected to electrochemical measurements. The evaluated parameter was the charge transfer resistance (R_ct_), obtained from electrochemical impedance spectroscopy (EIS) following the modification of the electrodes with the nanocomposite and biomolecules.

### 2.4. miRNA Biosensor Optimization and Calibration Curve

The optimization of probe concentrations ([anti-miRNA-29a] and [anti-miRNA-34a]) and their incubation time (time_inc_) on the surface of glassy carbon electrodes (GCEs) was carried out individually using a 3^2^ multivariate factorial design. The concentrations of the respective targets ([miRNA-29a] and [miRNA-34a] = 10 pM) and the hybridization time (time hib = 50 min) were kept constant throughout the experiments. The optimal condition for each biosensor was evaluated based on the percentage variation in charge transfer resistance (∆R_ct_) obtained from EIS analysis, using the following equation:

(1) $${∆R}_{ct}=\left(\frac{R_{ct\left(hyb\right)-}R_{ct\left(bios\right)}}{R_{ct\left(bios\right)}}\right)\times 100\%$$

where R_ct(bios)_ refers to the charge transfer resistance measured after incubation but prior to hybridization, and R_ct(hyb)_ corresponds to the value obtained after the hybridization step. All measurements were performed at room temperature (25 °C). Based on preliminary studies, the experimental levels used in the factorial design were: [anti-miR-29a] = 5 (−1), 10 (0) and 15 (+1) pM; [anti-miR-34a] = 1 (−1), 5 (0) and 10 (+1) pM; and for both probes time inc = 25 (−1), 50 (0) and 75 (+1) min. For the construction of the analytical curve, the hybridization efficiency was also evaluated using the ∆ratio method described by Bonanni and del Valle (2010) [31], which was applied to determine the R_ct_ variation associated with each concentration following the preparation of a new sensing platform. The calculation was based on the equation:

∆ratio = ∆s/∆p (2)

where ∆s = R_ct(hyb)_ − R_ct(blank)_ and ∆p = R_ct(bios)_ − R_ct(blank)_. Here, R_ct(blank)_ refers to the charge transfer resistance value obtained after modification of the electrode with the AuNPs–Si4DMAP^+^Cl^−^ nanohybrid. This approach takes into account the relative variations in R_ct_ values observed after platform renewal in each analysis.

The analytical performance of the proposed biosensor was evaluated by constructing a calibration curve using target miRNA solutions with concentrations ranging from 0.01 to 100 pM prepared in PBS buffer. The biosensor response was obtained by monitoring the variation in the ∆R_ct_ as a function of the miRNA concentration, allowing the determination of the linear detection range of the biosensor under controlled conditions. Subsequently, selected concentrations within this linear range were used to evaluate biosensor performance in diluted serum samples.

To evaluate the biosensor performance in biological samples, human serum diluted 1:10 in PBS was used as the analysis matrix. The control sample (0 pM) consisted of serum diluted 1:10 in PBS, with PBS added instead of the miRNA solution to maintain identical experimental conditions. Spiked samples were prepared by diluting the target miRNA solutions directly in serum previously diluted 1:10 with PBS at the selected concentrations.

### 2.5. Electrochemical Measurements

The characterization steps of the GCE and the construction of the biosensors were performed using the following techniques: cyclic voltammetry (CV) in the range from −0.3 V to −0.9 V; square wave voltammetry (SWV) in the range from −0.2 V to 1.0 V, with an amplitude of 0.02 V and frequency of 25 Hz; and electrochemical impedance spectroscopy (EIS) in the frequency range from 20 kHz to 100 mHz, employing a Randles equivalent circuit [R(Q[RW])]. All electrochemical techniques were carried out using a three-electrode system, with the modified GCE as the working electrode, Ag|AgCl as the reference electrode, and a platinum wire as the counter electrode, in an electrochemical cell containing 10 mL of 0.1 mol L^−1^ PBS buffer in the presence of the redox probe K_4_[Fe(CN)_6_]/K_3_[Fe(CN)_6_] at 5.0 mmol L^−1^ as the supporting electrolyte. At each stage of GCE modification, variations in peak currents (ip), anodic peak current (ipa), cathodic peak current (ipc), peak potentials and Rct values were observed. During biosensor construction, the immobilization steps of the anti-miRNA-29a and anti-miRNA-34a probes, as well as the hybridization with the target miRNA-29a and miRNA-34a sequences, respectively, were characterized by EIS. These measurements were performed with the GCE surfaces kept moist and rinsed with PBS at room temperature (25 °C), thereby ensuring that only miRNAs hybridized to the surface were detected. The matrix evaluation experiments were performed under the same experimental conditions.

## 3. Results

### 3.1. Si4DMAP^+^Cl^−^ and AuNPs-Si4DMAP^+^Cl^−^ Characterization

The synthesis of nanoparticles stabilized in silsesquioxane matrices has been reported in the literature since 2012 [23,32,33]. However, no studies have reported the use of silsesquioxane Si4DMAP^+^Cl^−^ as stabilizing agent for incorporation of AuNPs within its structure. It is assumed that, once formed, the AuNPs are embedded and stabilized within the disordered polymeric network of the silsesquioxane [29], through a combination of electrostatic and steric effects that prevent nanoparticle coalescence.

**Figure 2:**
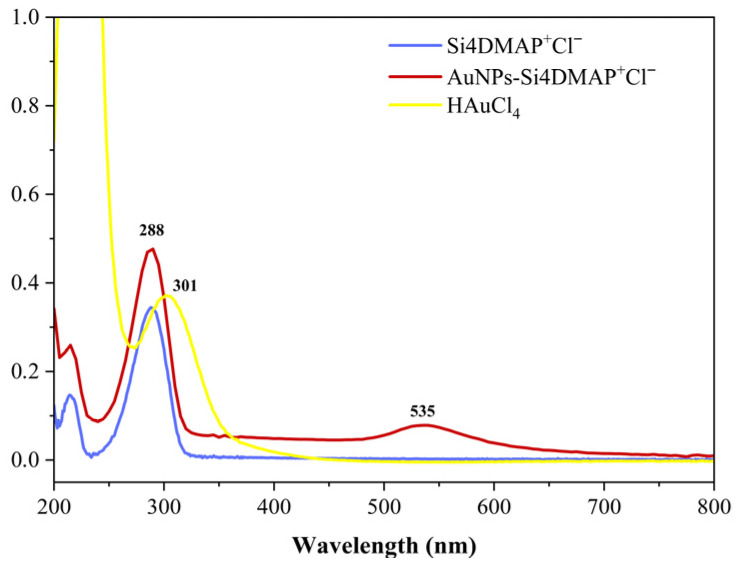
UV-Vis absorption spectra of the AuNPs–Si4DMAP^+^Cl^−^, Si4DMAP^+^Cl^−^ and HAuCl_4_ dispersions obtained in the range of 200 to 800 nm.

To investigate this incorporation, UV-Visible absorption spectra were recorded for the AuNPs–Si4DMAP^+^Cl^−^ nanohybrid (Figure 2) and compared with the spectra of the silsesquioxane polymer (Si4DMAP^+^Cl^−^) and the gold precursor salt (HAuCl_4_). The absorption band observed at 535 nm in the spectrum of AuNPs–Si4DMAP^+^Cl^−^ was attributed to the surface plasmon resonance (SPR) effect of the AuNPs. The absence of the 301 nm band, typically assigned to the ligand-to-metal charge transfer in HAuCl_4_ [34], confirms the reduction of AuCl_4_^−^ to metallic Au^0^. In the UV region of the Si4DMAP^+^Cl^−^ spectrum, a band at 288 nm is observed, corresponding to the π–π* transition of the pyridine ring [29], which was also present in the spectrum of the nanohybrid, indicating that this band was not shifted upon AuNPs incorporation.

**Figure 3:**
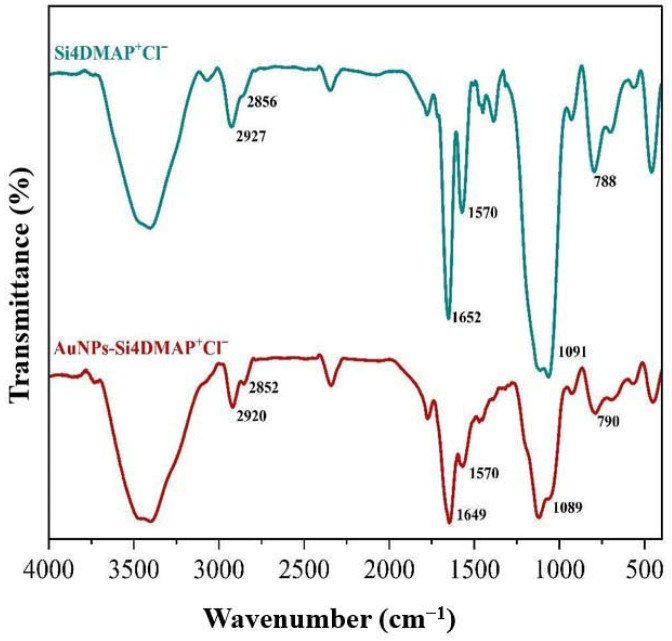
Infrared vibrational spectra for the Si4DMAP^+^Cl^−^ material and the AuNPs–Si4DMAP^+^Cl^−^ nanohybrid obtained in KBr.

The FTIR spectrum of Si4DMAP^+^Cl^−^ (Figure 3) shows characteristic absorption bands at 2927 and 2856 cm^−1^, attributed to asymmetric and symmetric Csp^3^–H stretching vibrations from methyl groups, as well as bands at 1652 cm^−1^ (C=C stretching) and 1570 cm^−1^ (C=N stretching), confirming the functionalization of the silsesquioxane with 4-DMAP [24]. These bands appear slightly shifted in the AuNPs–Si4DMAP^+^Cl^−^ spectrum, at 2920 and 2852 cm^−1^, and at 1649 and 1570 cm^−1^, respectively. Additionally, the asymmetric and symmetric stretching bands of the Si–O–Si groups observed at 1091 and 788 cm^−1^ in the Si4DMAP^+^Cl^−^ spectrum appeared at 1089 and 790 cm^−1^ with reduced intensity in the nanohybrid spectrum. These spectral changes are likely due to interactions between Au and the Si–O–Si framework, which affect the vibrational modes of the polyelectrolyte as a result of AuNP immobilization, as also observed in other studies involving AuNPs stabilized in ionic silsesquioxane-based polymers [34].

Zeta potential (ζ) analyses were carried out for both the AuNPs–Si4DMAP^+^Cl^−^ nanohybrid and the Si4DMAP^+^Cl^−^ polymer. Both materials exhibited good colloidal stability, with positive ζ-potential values of +43.0 mV and +34.0 mV, respectively, which was expected due to the presence of pyridinium groups in the silsesquioxane structure.

**Figure 4:**
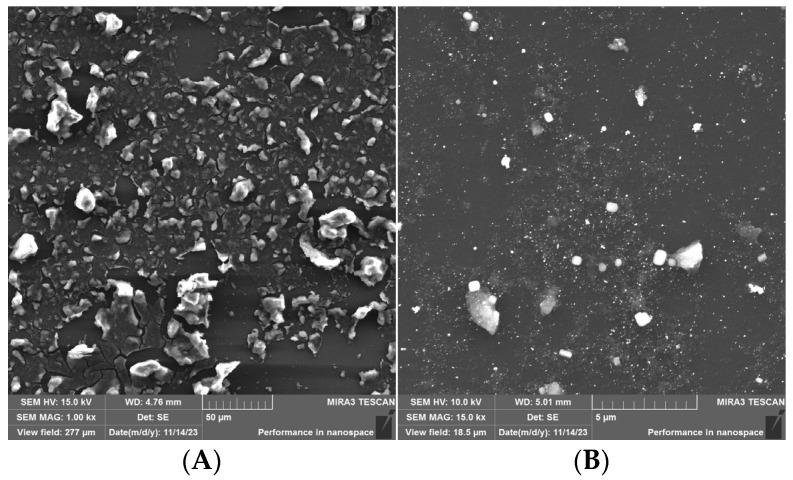
FEG-SEM images obtained for (**A**) Si4DMAP^+^Cl^−^ and (**B**) for the AuNPs–Si4DMAP^+^Cl^−^ nanohybrid.

Morphological characterization was performed by field-emission scanning electron microscopy. The SEM images of Si4DMAP^+^Cl^−^ (Figure 4A) display a uniformly rough surface. In contrast, the images of the nanohybrid (Figure 4B) revealed the presence of clusters at specific regions and a higher number of small particles distributed throughout the glassy carbon surface, which are attributed to metallic nanoparticles.

**Figure 5:**
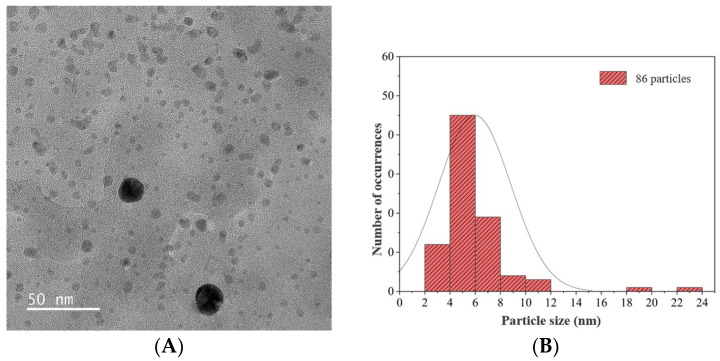
(**A**) Transmission Electron Microscopy (TEM) obtained for AuNPs–Si4DMAP^+^Cl^−^ with a scale bar of 50 nm and (**B**) particle size distribution histogram of the AuNPs generated from the TEM image of 86 particles, showing the measured diameters.

TEM was also employed to assess the size and morphology of the nanoparticles (Figure 5A). From TEM images, a total of 86 particles were measured using ImageJ software version 1.54g, and the resulting particle uniform in size distribution is shown in Figure 5B.

The particles exhibited a spherical shape, with an average diameter of 6 nm, which is significantly smaller than the average size observed by Dynamic Light Scattering (DLS). This discrepancy can be attributed to the fact that DLS measures the hydrodynamic radius of the AuNPs, including the surrounding water layers and the Si4DMAP^+^Cl^−^ stabilizer, while TEM relies on the transmission of the incident electron beam through the sample.

### 3.2. Electrochemical Characterization of GCE, GCE/Si4DMAP^+^Cl^−^ and GCE/AuNPs-Si4DMAP^+^Cl^−^

In order to evaluate the effect of AuNPs on the electrochemical response, electrochemical characterizations were performed for the unmodified GCE, the GCE modified only with the silsesquioxane (Si4DMAP^+^Cl^−^) and with the nanohybrid AuNPs–Si4DMAP^+^Cl^−^. At each modification step, the electrochemical techniques of CV, SWV and EIS were carried out in PBS containing the redox probe K_4_[Fe(CN)_6_]/K_3_[Fe(CN)_6_] at 5.0 mmol L^−1^, in order to compare the responses obtained from each platform using the three techniques.

**Figure 6:**
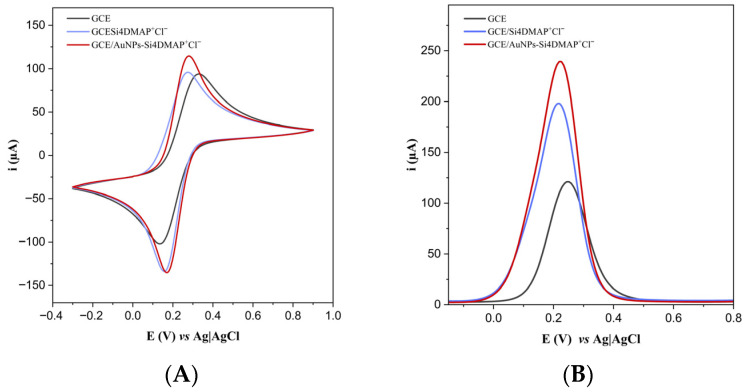
(**A**) Cyclic voltammograms (scan rate of 100 mV s^−1^) and (**B**) square wave voltammograms (applied potential range: −0.2 V to 1.0 V, amplitude of 0.02 V and frequency of 25 Hz) for GCE, GCE/Si4DMAP^+^Cl^−^ and GCE/AuNPs–Si4DMAP^+^Cl^−^ obtained in 0.1 mol L^−1^ PBS containing 5.0 mmol L^−1^ K_4_[Fe(CN)_6_]/K_3_[Fe(CN)_6_].

The CV voltammograms (Figure 6A) show that incorporation of the AuNPs–Si4DMAP^+^Cl^−^ nanohybrid onto the GCE surface led to significantly higher anodic (I_pa_) and cathodic (I_pc_) peak currents compared to both the bare GCE and the GCE/Si4DMAP^+^Cl^−^, although the latter also caused a substantial increase in I_pc_. The increase in peak currents of the GCE/Si4DMAP^+^Cl^−^ is attributed to the positively charged structure of the silsesquioxane, which enhances electrostatic interactions with the negatively charged redox couple [Fe(CN)_6_]^4−^/[Fe(CN)_6_]^3−^. It was also noted that the GCE/AuNPs–Si4DMAP^+^Cl^−^ exhibited the lowest peak-to-peak potential difference (ΔE_p_) and the highest values of I_pa_ and I_pc_ among the studied electrodes. The shift in both anodic and cathodic peak potentials (E_pa_ and E_pc_) indicates that this surface facilitates electron transfer processes, highlighting the ability of the nanohybrid to promote redox reactions an effect attributed to the electrocatalytic activity of the incorporated AuNPs [34,35].

Supporting the observations from CV, the SWV results (Figure 6B) further confirmed the increase in peak currents resulting from the incorporation of both the inorganic polymer and the nanohybrid, as SWV where the nonfaradaic current is minimized [36]. A 63.7% increase in peak current (I_p_) was observed upon the addition of Si4DMAP^+^Cl^−^ to the GCE, and for the GCE/AuNPs–Si4DMAP^+^Cl^−^ the current increased by 97.9% compared to the bare GCE. This enhancement is attributed not only to electrostatic interactions but also to the presence of AuNPs, which promote electron transfer between the redox species and the electrode surface [31], thereby improving the electrochemical signal due to the aforementioned catalytic properties.

**Figure 7:**
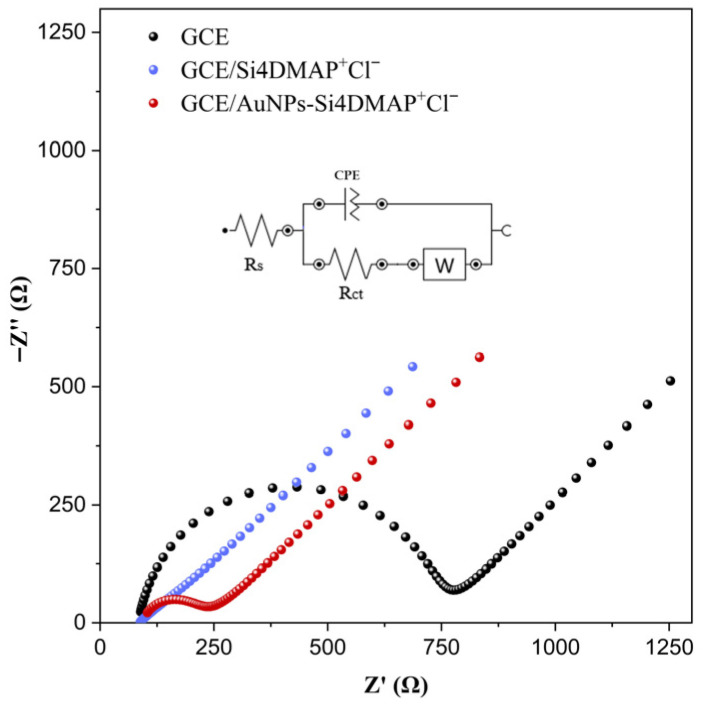
Nyquist plots obtained from EIS measurements for GCE, GCE/Si4DMAP^+^Cl^−^ and GCE/AuNPs–Si4DMAP^+^Cl^−^ in 0.1 mol L^−1^ PBS containing 5.0 mmol L^−1^ K_4_[Fe(CN)_6_]/K_3_[Fe(CN)_6_], in the frequency range from 20 kHz to 100 mHz.

In EIS, R_ct_ parameter reflects the resistance to electron transfer at the electrode/solution interface. The Nyquist plots obtained from EIS analysis (Figure 7) exhibit two distinct regions: a linear part corresponding to ion diffusion and a semicircular portion indicating electron transfer limitations [37].

The presence of Si4DMAP^+^Cl^−^ and AuNPs–Si4DMAP^+^Cl^−^ significantly favored the charge transfer, resulting in a notable reduction in R_ct_ values compared to the unmodified GCE. The bare GCE exhibited a high R_ct_, which decreased significantly from 657 Ω to 382 Ω after modification with Si4DMAP^+^Cl^−^. This behavior can be attributed to the electrostatic attraction between the positively charged groups in the material structure and the negatively charged [Fe(CN)_6_]^4−^/[Fe(CN)_6_]^3−^ redox couple. However, a much more pronounced decrease in R_ct_ was observed after modification of the GCE with the nanohybrid, reaching 159 Ω upon incorporation onto the electrode surface, which is associated with enhanced electrocatalytic processes promoted by the AuNPs. Therefore, the incorporation of AuNPs in the biosensor architecture is essential both to improve the electrochemical performance and to enable the immobilization of oligonucleotides via covalent anchoring of thiolated oligonucleotides through strong Au–S bond formation.

**Figure 8:**
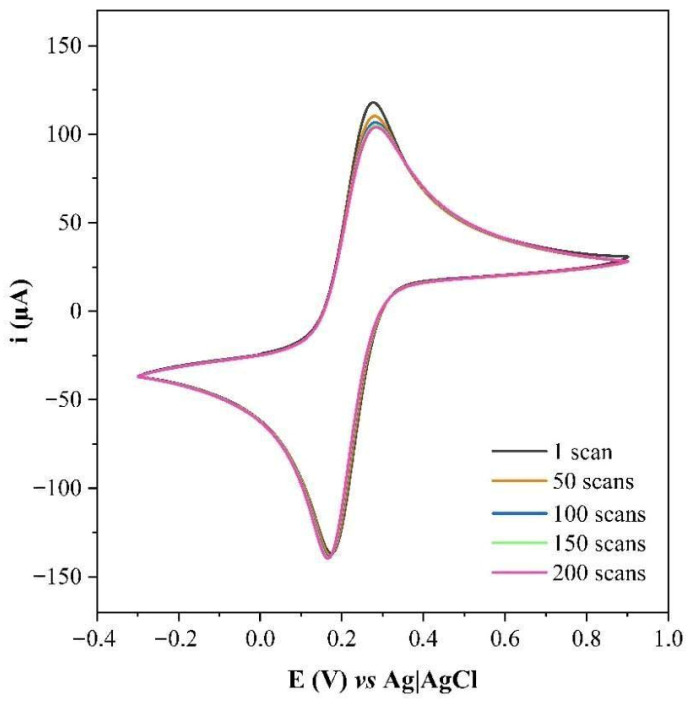
Cyclic voltammograms obtained for GCE/AuNPs–Si4DMAP^+^Cl^−^ to monitor its stability after 200 potential scans in 0.1 mol L^−1^ PBS containing 5.0 mmol L^−1^ K_4_[Fe(CN)_6_]/K_3_[Fe(CN)_6_] (scan rate of 100 mV s^−1^).

CV was also used to assess the stability of the GCE/AuNPs–Si4DMAP^+^Cl^−^ by performing 200 consecutive scans. The voltammograms obtained (Figure 8) showed no significant variation in E_p_ or I_p_ values, with only a slight decrease in I_p_, likely due to minor leaching of the material.

**Figure 9:**
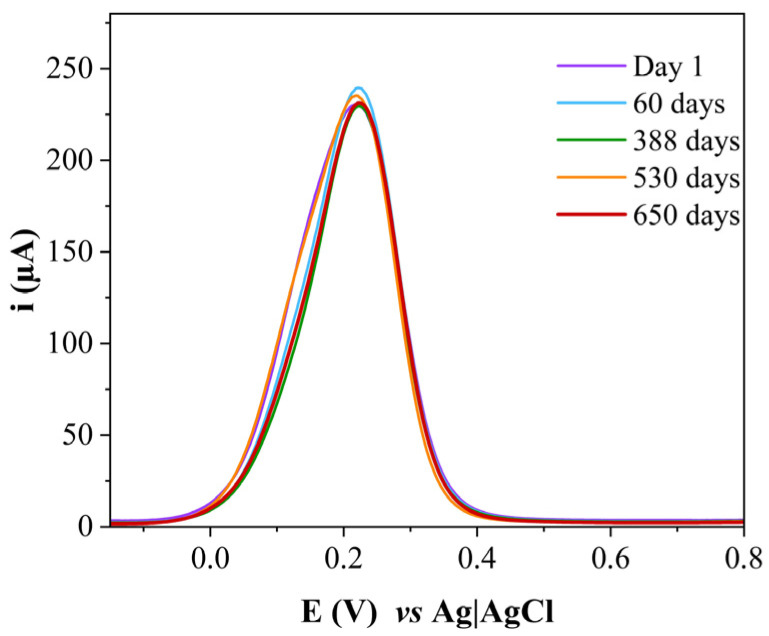
Square wave voltammograms obtained for GCE/AuNPs–Si4DMAP^+^Cl^−^ monitored over a period of 650 days from the date of its synthesis (range from −0.2 V to 1.0 V, amplitude of 0.02 V and frequency of 25 Hz).

This minimal loss suggests that the nanohybrid remained stably anchored on the electrode surface, especially considering that no additional anchoring strategies were employed and the total time of the electrochemical measurements was much shorter than the time that the platform remained immersed in solution during the scans. The long-term electrochemical stability of the AuNPs–Si4DMAP^+^Cl^−^ nanohybrid was also monitored via SWV over several months. On each day of measurement, the electrode was cleaned and a new platform was constructed before data collection. Figure 9 shows the voltammograms from the first day after synthesis up to day 650. The nanohybrid maintained consistent electrochemical behavior throughout this period, as the current values remained within the range observed in the early days, with a maximum variation of only 10 µA in I_p_ and no shift in peak potentials. These results confirm the excellent stabilization of the AuNPs within the silsesquioxane matrix, which effectively prevented the nanoparticle coalescence.

### 3.3. Electrochemical Characterization and Optimization of the miRNA Biosensor

During the optimization stage, the concentrations of the anti-miRNA-29a and an-ti-miRNA-34a probes, as well as the immobilization time, were evaluated. The parameter used to determine the optimal conditions was the highest ∆Rct value obtained after the hybridization process between the probe and the target. For each assay, the electrode was cleaned and a new sensing platform was assembled. The investigation of optimal probe immobilization conditions is of great importance, as target molecule detection is directly influenced by factors such as the homogeneity and density of the monolayer formed on the GCE surface by the immobilized anti-miRNA strands [38]. If the surface becomes saturated with immobilized probes, the sensitivity of the system may be compromised due to the limited accessibility of the target molecules to the probes, thereby hindering hybridization.

A 3^2^ factorial design (Table S1) was employed to investigate the effects of probe con-centration and immobilization time in order to establish the optimal experimental conditions for the two selected anti-miRNAs, while keeping the target concentration constant at 10 pM. Based on the ∆Rct results, it was observed that longer probe immobilization times were not favorable for either miRNA, especially when combined with increased probe concentration. The decrease in ∆R_ct_ values at higher probe concentrations may be attributed to an excessive amount of immobilized anti-miRNA strands on the electrode surface, which can hinder hybridization with the target sequences. Regarding the immobilization time, shorter time durations may promote the formation of more organized and stable arrangements on the GCE/AuNPs–Si4DMAP^+^Cl^−^ surface.

Analysis of the results showed that the highest ∆Rct values for both miRNAs were obtained under the conditions [anti-miRNA-29a] and [anti-miRNA-34a] = 10 pM and immobilization time = 25 min, even though the levels employed in the factorial designs were different due to previous studies. Reducing the immobilization time is also advantageous for decreasing the overall preparation time of the sensing platform. These parameters were therefore adopted for the construction of the analytical calibration curves, maintaining the hybridization time at 50 min. Additionally, for both the analytical curve construction and real sample analysis, it was necessary to block non-binding sites on the GCE surface with BSA after probe immobilization. This step aims to minimize nonspecific adsorption of molecules on the modified GCE surface [38,39,40].

### 3.4. Analytical Curve and Biosensor Performance in Serum

**Figure 10:**
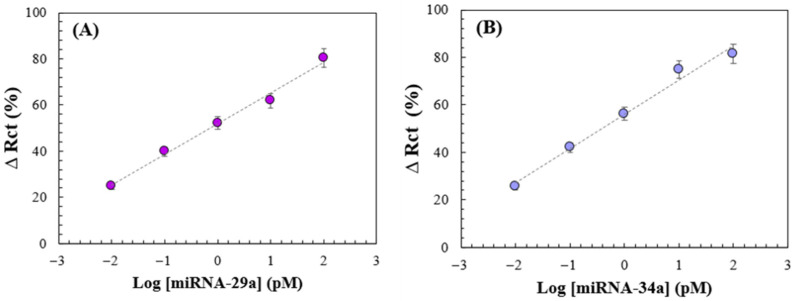
Analytical curves obtained based on the mean ∆R_ct_ values (presented in Table S2) for (**A**) GCE/AuNPs–Si4DMAP^+^Cl^−^/anti-miRNA-29a/BSA and (**B**) GCE/AuNPs–Si4DMAP^+^Cl^−^/anti-miRNA-34a/BSA after hybridization with different target concentrations from 0.01 to 100 pM.

The analytical calibration curve (Figure 10A,B) was constructed under the optimized immobilization conditions for anti-miRNA-29a and anti-miRNA-34a, with the addition of BSA, keeping the hybridization time of the target miRNAs fixed (time hib = 50 min) and varying their concentrations through serial dilutions in ultrapure water.

**Figure 11:**
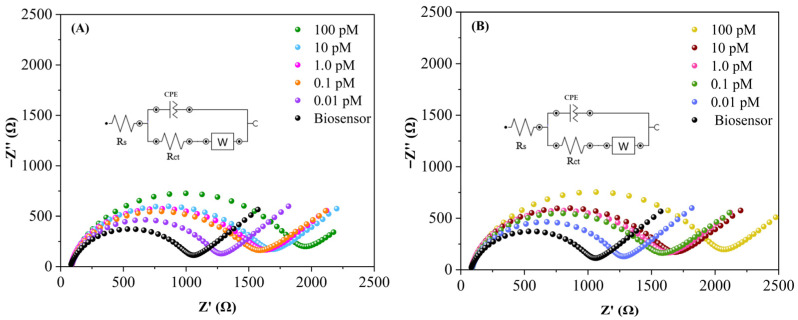
Nyquist plots obtained from EIS for (**A**) GCE/AuNPs–Si4DMAP^+^Cl^−^/anti-miRNA-29a/miRNA-29a and (**B**) GCE/AuNPs–Si4DMAP^+^Cl^−^/anti-miRNA-34a/miRNA-34a at different concentrations. Measurements performed in 0.1 mol L^−1^ PBS containing 5.0 mmol L^−1^ K_4_[Fe(CN)_6_]/K_3_[Fe(CN)_6_], over the frequency range of 20 kHz to 100 mHz.

The concentration range began with the lowest values of [miRNA-29a] and [miRNA-34a] and extended to a maximum of 100 pM for both targets. The response for each concentration was analyzed based on ∆Rct monitored via EIS measurements (Figure 11A,B). Hybridization at different concentrations was also evaluated through the relative variation in Rct values after biosensor renewal for each concentration level, expressed as the ∆ratio (as defined in Equation (2)). According to Bonanni and del Valle (2010) [31], the occurrence of the hybridization process is confirmed when ∆ratio values exceed 1. The combined use of ∆Rct and ∆ratio provided a more reliable assessment of the hybridization efficiency and allowed for better evaluation of platform performance.

For samples without the presence of miRNAs ([miRNA] = 0 pM), the charge transfer resistance showed no significant changes, as demonstrated by mean ∆R_ct_ values close to 1. In contrast, as the concentrations of miRNA-29a and miRNA-34a increased, a proportional increase in mean ∆R_ct_ values was observed (Table S2). An increase of 223.8% in R_ct_ values was observed for miRNA-29a and 219.5% for miRNA-34a between the lowest concentration and the maximum concentration of 100 pM. This increase is attributed to a higher number of hybridized target molecules on the electrode surface, which forms a more substantial barrier to the electron transfer as concentration rises. A similar trend was observed in ∆ratio values which also increased proportionally with target concentration, confirming that more miRNA molecules were hybridized at each concentration increment for both targets. This analysis demonstrated that R_ct_ values remained proportional, even when considering variations introduced by the renewal of the electrode surface with the nanohybrid for each measurement, indicating that the response of the electrochemical platform remained linear across the concentration range studied.

When plotting ∆R_ct_ values against the logarithm of miRNA concentration, a linear correlation was obtained for each miRNA, yielding the following regression equations: miRNA-29a = 13.327 log[miRNA-29a] + 51.84 (R = 0.9912) and miRNA-34a = 14.462 log[miRNA-34a] + 55.99 (R = 0.9840). Both biosensors were capable of detecting changes in miRNA concentrations across a range of 0.01 to 100 pM, demonstrating the sensitivity of the platform. This was further supported by ANOVA statistical analysis, which yielded a *p*-value of approximately 0.0006, indicating a 99% confidence level for both miRNA-29a and miRNA-34a.

Based on the results from the analytical curves the limit of detection (LOD) and limit of quantification (LOQ) were calculated for both biosensors in accordance with ANVISA guidelines [36]. The LOD for miRNA-29a and miRNA-34a was 1.79 pM and 2.21 pM, respectively. Compared with studies available in the literature on the electrochemical detection of miRNA-29a and miRNA-34a (see Table 1), the biosensor developed in this work exhibited a lower LOD than most platforms described to date. Additionally, the LOQ was determined to be 5.87 pM for miRNA-29a and 11.01 pM for miRNA-34a.

Circulating levels of miR-29a and miR-34a in peripheral biofluids have been widely investigated in Alzheimer’s disease and clinically relevant concentrations in blood samples typically range from low nanomolar (nM) to femtomolar (fM) levels, although most studies report relative expression rather than absolute concentrations. Beyond these specific targets, the overall behavior of circulating miRNAs has been extensively investigated, with studies consistently demonstrating their stability and detectability in biological fluids. A recent study by Katayama et al. (2025) [43] demonstrated the high stability of multiple circulating miRNAs in plasma and serum under different storage conditions using RT-qPCR. These findings support the feasibility of using circulating miRNAs as minimally invasive biomarkers, while also reinforcing the need for highly sensitive analytical platforms capable of detecting low-abundance targets in complex biological matrices. However, in this study, miRNA expression levels are reported as counts per million (CPM), which reflect relative abundance rather than absolute concentration.

Similarly, in a study conducted by Gevaert et al. (2018) [44], which profiled circulating miRNAs in plasma using qPCR, 233 out of 377 investigated miRNAs exhibited quantification cycle (Cq) values ≤ 35, indicating that a substantial proportion of circulating miRNAs are detectable within the reliable amplification range. Therefore, most clinical studies report miRNA levels using relative quantification approaches, such as fold change, Cq values, or area under the curve (AUC), rather than absolute concentrations expressed in molar units (e.g., aM or pM). Despite the lack of absolute quantification, these approaches remain highly valuable for identifying disease-associated expression patterns. Nevertheless, further studies are required to determine absolute concentrations and enable direct comparison with the detection limits of emerging biosensing platforms.

In this context, considering the reported downregulation of miR-29a and miR-34a in Alzheimer’s disease, it is worth highlighting that, in all experiments, the proposed device was constructed using minimal volumes of the AuNPs–Si4DMAP^+^Cl^−^ nanohybrid, of the anti-miRNA probes and of the target miRNAs, only 5.0 µL of each sample. Furthermore, the probes proved to be effective even at the low concentration defined by the factorial design ([anti-miR-29a] and [anti-miR-34a] = 10 pM), demonstrating the economical use of materials in the modification process. Additionally, platform fabrication requires only a few simple fabrication steps. The relatively short assay time further highlights the potential of the proposed system for practical and rapid biosensing applications. An important advantage of the proposed biosensor lies in the simplicity of its sensing strategy. The analytical response is obtained through a straightforward process involving only the modification of the electrode with the AuNP–silsesquioxane nanohybrid, followed by probe immobilization and hybridization with the target miRNAs. In contrast, several previously reported electrochemical miRNA biosensors rely on more complex sensing schemes, frequently involving enzymatic amplification reactions, catalytic hairpin assembly, or multiple DNA nanostructures to enhance the signal. These additional amplification strategies generally require multiple preparation steps and longer assay procedures. Therefore, the proposed platform represents a simpler and more direct approach while still providing sensitive detection of target miRNAs.

The advantages of the AuNP-silsesquioxane nanohybrid are demonstrated by its excellent stabilization and its applicability for detecting two different miRNAs using the same platform, showing effectiveness for both targets. Silsesquioxane contributes to probe immobilization through its positive charges, as confirmed by additional tests with silsesquioxane alone (without AuNPs), which still promoted anti-miRNA immobilization at lower concentrations, though less effectively. Furthermore, the organic functional groups attached to the SSQ inorganic backbone provide a versatile interface for bonding with various compounds and biomolecules. Also, compared to the cited methods, which utilized HAuCl_4_ concentrations ranging from 25 to 100 mM, our study employed a significantly lower concentration of 2.5 mM. Moreover, silsesquioxane provides good solubility, high stability and the formation of small, uniform nanoparticles.

**Figure 12:**
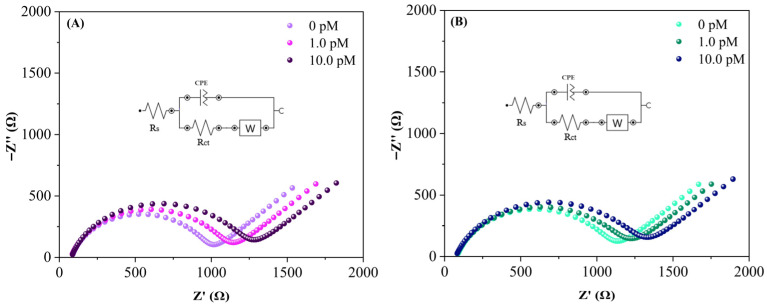
Nyquist plots obtained for the detection of (**A**) miRNA-29a and (**B**) miRNA-34a in serum diluted 1:10 in PBS after incubation with different concentrations of target miRNA (0, 1 and 10 pM). Measurements performed in 0.1 mol L^−1^ PBS containing 5.0 mmol L^−1^ K_4_[Fe(CN)_6_]/K_3_[Fe(CN)_6_], over the frequency range of 20 kHz to 100 mHz.

In order to perform a preliminary evaluation of the applicability of the proposed biosensor in a complex biological matrix, detection experiments were also performed in serum. For this purpose, serum samples were spiked with the target miRNAs at concentrations of 1 and 10 pM, while a non-spiked serum sample was used as a blank (0 pM). These concentrations were selected within the linear range (0.01–100 pM) previously stablished during the calibration experiments. The assays performed in serum samples were also analyzed by EIS and the variation in the electrochemical response can be observed in the Nyquist plots (Figure 12A,B), which show changes in the semicircle diameter, as well as in the corresponding ΔR_ct_ values summarized in Table 2.

The obtained ∆Rct followed the same trend observed in the aqueous solution system, confirming that the proposed platform is capable of detecting the target miRNAs even in the presence of complex biomolecular components. Even under these conditions, the biosensor maintained a concentration-dependent response for the target miRNAs. Although diluted, the serum matrix still contains a variety of biomolecules such as proteins, salts and metabolites, representing a considerably more complex environment than the buffer solution used for calibration. Evaluating the biosensor response under these conditions provides a more realistic assessment of its performance in biological samples. These results further support the applicability of the proposed platform and highlight its competitive performance compared with previously reported miRNA electrochemical sensors.

## 4. Discussion

This is the first study to report the incorporation of AuNPs into the structure of Si4DMAP^+^Cl^−^ since its synthesis. The resulting nanohybrid, AuNPs–Si4DMAP^+^Cl^−^, exhibited excellent suspension stability, verified by ζ, and characteristic nanoscale dimensions, observed by DLS and TEM techniques. Its stability was further confirmed through electrochemical measurements performed over a period exceeding 600 days, due to the silsesquioxane matrix in preventing AuNP aggregation and contributing to long-term stabilization. The incorporation of the nanohybrid onto the GCE surface improved electrochemical response and enhanced charge transfer processes, as well as effective electrocatalysis of the redox probe employed. These findings demonstrate that AuNPs–Si4DMAP^+^Cl^−^ have a potential for the development of stable electrochemical biosensors and other long-term analytical devices.

The nanohybrid AuNPs–Si4DMAP^+^Cl^−^ proved to be efficient in immobilizing anti-miRNA-29a and anti-miRNA-34a probes on the GCE surface, likely due to the presence of AuNPs and the positively charged silsesquioxane, which promote biomolecule interaction. This efficiency was evidenced by a decrease in anodic peak current and an increase in resistance of charge transfer, as observed by SWV and EIS analyses, respectively. After optimization of parameters of probe concentration and incubation time, the biosensors demonstrated low detection limits, comparable to those reported in previous studies involving the electrochemical detection of the same miRNAs. In addition to the favorable characteristics of the biosensor, the novelty of this work is highlighted by the synthesis of an unprecedented nanohybrid and its application in this biosensing platform. This performance is mainly attributed to the presence of the silsesquioxane-based polyelectrolyte, whose positively charged framework promotes the stabilization of the AuNPs, facilitates probe immobilization and contributes to the improved sensitivity of the developed biosensor. Although further investigations will be necessary to fully validate its analytical performance, these results demonstrate the potential applicability of this nanohybrid for developing electrochemical biosensors capable of detecting Alzheimer’s disease-associated miRNAs in serum samples. Moreover, the proposed biosensing platform is simple and can be fabricated through an easily reproducible process, facilitating its potential implementation in miniaturized devices. In conclusion, this platform demonstrates the ability to sensitively detect Alzheimer’s disease-associated miRNAs, such as miR-29a and miR-34a, and offers a minimally invasive, complementary approach to conventional diagnostic methods, with the potential to support earlier detection before symptom onset and inform clinical decision-making. Future studies should focus on integrating the platform into specific biosensing architectures and evaluating its performance with real biological samples.

## Figures and Tables

**Table 1:** Studies reported in the literature on the electrochemical detection of miRNA-29a and miRNA-34a associated with Alzheimer’s disease.

Target	Biosensor	Electrochemical Technique	LOD (nM)	Reference
^1^ miRNA-29a	SPCE/AuNPs	SWV	0.15	[20]
^2^ miRNA-34a	GO-CA-PGE	EIS	72	[21]
^1^ miRNA-34a	SPCE/AuNPs	EIS	9.4 × 10^−8^	[22]
^3^ miRNA-34a	ZNA/MBs/PGE	DPV	0.121	[41]
^4^ miRNA-34a	SPE-CNF	DPV	1.53	[42]
miRNA-29a	GCE/AuNPs-Si4DMAP^+^Cl^−^	EIS	1.79 × 10^−3^	This work
miRNA-34a			2.21 × 10^−3^	

^1^—SPCEs modified with AuNPs. ^2^—Pencil graphite electrode (PGE) chemically functionalized with covalent agents (CA) and modified with GO. ^3^—ZIP nucleic acid (ZNA) used as a probe for hybridization with miRNA-34a, applied in the modification of a PGE after magnetic separation using magnetic beads (MBs). ^4^—Screen-printed electrodes modified with carbon nanofibers (SPE-CNF). All concentrations were converted to nM to facilitate comparison. Values originally reported in µg/mL were converted to molar concentration considering the approximate molecular weight of miRNA-34a.

**Table 2:** ΔRct values obtained from EIS measurements after incubation with different concentrations of target miRNA-29a e miRNA-34a in serum diluted 1:10 in PBS. The variation in ΔRct was calculated with relative to the probe-modified electrode.

[miRNA-29a] in diluted serum 1:10 (pM)	∆R_ct_ (%)
0	48.75
1.0	55.01
10.0	81.00
**[miRNA-34a] in diluted serum 1:10 (pM)**	**∆R_ct_ (%)**
0	53.30
1.0	62.55
10.0	74.90

## Data Availability

The data that support the findings of this study are included in the article and further inquiries can be directed to the corresponding author, due to privacy restrictions.

## Supplementary Materials

The following supporting information can be downloaded at: https://www.mdpi.com/article/10.3390/s26072089/s1, Table S1—Design matrix of the 3^2^ factorial planning used for the optimization of the variables involved in the biosensor assembly step. The variables studied were: [anti-miRNA-29a] = 5 (−), 10 (0), and 15 pmol µL^−1^ (+); [anti-miRNA-34a] = 1 (−), 5 (0), and 10 pmol µL^−1^ (+); and incubation time for both = 25 (−), 50 (0), and 75 min (+); Table S2—Mean ∆Rct values and corresponding standard deviations obtained in duplicate for the detection assay of miRNAs 29a and 34a by EIS at different concentrations.
