# Supplementary material for: Electrochemical Detection of miR-29a and miR-34a Using AuNPs Immobilized by a Silsesquioxane Polyelectrolyte: Potential Early Alzheimer’s Disease Biomarkers Detection
Source: Sensors (Basel). 2026 Mar 27;26(7):2089. doi: 10.3390/s26072089 (PMC13074746; doi:10.3390/s26072089)
Supplement: Supplementary file 1 [file sensors-26-02089-s001.zip › sensors-4202480-supplementary.pdf]

Supplementary material

Table S1 - Design matrix of the 3^2 factorial planning used for the optimization of the variables involved in the biosensor assembly step. The variables studied were: [anti-miRNA-29a] = 5 (−), 10 (0), and 15 pM (+); [anti-miRNA-34a] = 1 (−), 5 (0), and 10 pM (+); and incubation time for both = 25 (−), 50 (0), and 75 min (+).

**Assay**	**[anti-miRNA-29a]**	**$Time_{inc}$**	**ΔR_{ct} (%)**
1	-	-	70,3
2	-	+	25,1
3	-	0	77,5
4	0	-	**87,2**
5	0	0	39,4
6	0	0	33,2
7	0	0	32,1
8	0	+	64,6
9	+	-	60,2
10	+	0	47,2
11	+	+	57,4
Assay	**[anti-miRNA-34a]**	**$Time_{inc}$**	**ΔR_{ct} (%)**
1	-	-	118,1
2	-	+	98,5
3	-	0	74,7
4	0	-	94,5
5	0	0	41,6
6	0	0	41,5
7	0	0	37,3
8	0	+	60,7
9	+	-	**122,6**
10	+	0	75,7
11	+	+	46,0

Table S2 – Mean ΔR_{ct} values and corresponding standard deviations obtained in duplicate for the detection assay of miRNAs 29a and 34a by EIS at different concentrations.

**[miRNA-29a] (pM)**	**ΔR_{ct} (%)**	**SD**	Δ_{ratio}
0	0,34	0,321	1,01
0,01	24,82	0,533	1,29
0,1	40,09	0,369	1,44
1	52,26	0,145	1,59
10	61,96	2,469	1,72
100	80,37	0,261	1,90
**[miRNA-34a] (pM)**	**ΔR_{ct} (%)**	**SD**	**Δ_{ratio}**
0	1,10	0,844	1,01
0,01	25,48	0,171	1,30
0,1	42,04	0,881	1,45
1	56,22	0,085	1,62
10	74,80	3,51	1,85
100	81,41	1,170	1,94
